# Randomized phase 3 study of elotuzumab for relapsed or refractory multiple myeloma: ELOQUENT-2 Japanese patient subanalysis

**DOI:** 10.1038/bcj.2017.18

**Published:** 2017-03-10

**Authors:** K Suzuki, K Sunami, K Ohashi, S Iida, T Mori, H Handa, K Matsue, M Miyoshi, E Bleickardt, M Matsumoto, M Taniwaki

**Affiliations:** 1 Department of Hematology, Japanese Red Cross Medical Center, Tokyo, Japan; 2 Department of Hematology, National Hospital Organization Okayama Medical Center, Okayama, Japan; 3 Hematology Division, Tokyo Metropolitan Cancer and Infectious Diseases Center Komagome Hospital, Tokyo, Japan; 4 Department of Hematology and Oncology, Nagoya City University Hospital, Nagoya, Japan; 5 Division of Hematology, Department of Medicine, Keio University School of Medicine, Tokyo, Japan; 6 Integrative Center of Internal Medicine, Division of Hematology, Gunma University Hospital, Gunma, Japan; 7 Department of Internal Medicine, Kameda Medical Center, Kamogawa, Japan; 8 Bristol-Myers Squibb K.K, Tokyo, Japan; 9 Oncology Clinical Development, Bristol-Myers Squibb, Princeton, NJ, USA; 10 Department of Hematology, National Hospital Organisation, Shibukawa Medical Center, Shibukawa, Japan; 11 Department of Hematology, University Hospital, Kyoto Prefectural University of Medicine, Kyoto, Japan

Elotuzumab is a humanized immunoglobulin G1 immunostimulatory monoclonal antibody targeted against signaling lymphocytic activation molecule F7 (SLAMF7).^1^ A phase 1 clinical study (NCT01241292) of elotuzumab in combination with lenalidomide and dexamethasone (ELd) in six patients with relapsed/refractory multiple myeloma (RRMM) was conducted in Japan. The study reported an acceptable safety and tolerability profile and an overall response rate of 83% (5/6).^2^ ELOQUENT-2 (NCT01239797) is a phase 3, international, randomized clinical trial that investigated the efficacy and safety of ELd compared with lenalidomide/dexamethasone (Ld) in patients with RRMM who had received one to three previous therapies.^3^ Patients were randomized 1:1 to ELd (*n*=321) or Ld (*n*=325). Asian patients comprised 10% (64/646) of the overall population, of which 94% (60/64) were enrolled from Japan.

We performed a subanalysis to assess the efficacy and safety of ELd in the Japanese population from ELOQUENT-2, to determine whether results are aligned with those from the global study population. Coprimary endpoints were progression-free survival and overall response rate, assessed by an independent review committee. Further details of the study design can be found in the Supplementary Methods. Sixty patients from Japan were enrolled between October 2011 and September 2012: 31 randomized to the ELd group and 29 to the Ld group (Supplementary Table S1). At the time of data cutoff (October 2014) with follow-up of 2 years, 17 patients were still on treatment (ELd, *n*=12; Ld, *n*=5). The most common reason for discontinuation was disease progression. Eighty-four percent (26/31) of ELd patients tolerated ⩾90% of the planned elotuzumab dose. The median (95% confidence interval (CI)) progression-free survival was 22.2 (17.5–not estimated (NE)) months in the ELd group and 18.5 (11.1–21.2) months in the Ld group. The hazard ratio (HR) was 0.51 (95% CI: 0.25–1.06), representing a 49% reduction in the risk of disease progression or death in patients treated with ELd. The rate (95% CI) of progression-free survival in the ELd and Ld groups, respectively, was 74% (55–86%) and 66% (45–80%) at 1 year, and 48% (29–65%) and 18% (7–34%) at 2 years (Figure 1). There was no significant difference in overall response rate (95% CI) between treatment groups: 84% (66–95%) in the ELd group versus 86% (68–96%) in the Ld group (stratified common odds ratio 0.68 (0.16–2.90); Supplementary Table S2). A complete response or better was observed in four patients in the Ld group, but the rate of complete response or better in the ELd group may have been underestimated due to the interference of elotuzumab on serum protein electrophoresis and immunofixation assays.^4^

At the interim analysis of overall survival (October 2015), with follow-up of 3 years, 40% (24/60) of patients had died: 39% (12/31) in the ELd group and 41% (12/29) in the Ld group. Median overall survival was not reached. The interim HR (95% CI) for overall survival was 0.81 (0.35–1.87). One-year survival rates (95% CI) among ELd and Ld patients, respectively, were 100% (NE–NE) and 97% (78–100%), 2-year survival rates were 90% (73–97%) and 86% (67–94%), and 3-year survival rates were 68% (48–81%) and 64% (44–79%).

Although there are some limitations to this subpopulation analysis—the small sample and patient demographics, including the greater number of patients with high-risk cytogenetic profiles del(17p) and t(4;14) in the Ld group—ELd demonstrated efficacy in the Japanese cohort of ELOQUENT-2, similar to the high response rates and durable responses observed in the Japanese phase 1 study.^2^ Drug-related adverse events (AEs) of any grade were reported in 97% (30/31) of patients in the ELd group and 97% (28/29) in the Ld group (Supplementary Table S3). There were more non-hematological grade 3 or 4 AEs in the ELd group (94%) than in the Ld group (76%); the most common (⩾15%) included cataract (ELd 19% and Ld 14%) and pneumonia (ELd 19% and Ld 3%). Hematological AEs were similar between the ELd and Ld groups, except for grade 3 or 4 lymphopenia, which was more frequent in the ELd group (Supplementary Table S3). Serious AEs, regardless of relationship to treatment, were reported for 81% of patients in the ELd group and 62% in the Ld group. The most common serious AEs (⩾10% in any arm) were pneumonia and cataract (Supplementary Table S4). AEs leading to discontinuation of ⩾1 study drug occurred in 16% of patients in the ELd group versus 14% in the Ld group. Grade 3 or 4 AEs led to discontinuation of elotuzumab in 16% of ELd patients, whereas grade 3 or 4 AEs led to discontinuation in 7% of Ld patients. The most frequently reported cause of death was disease progression; one death in the ELd group was caused by myelodysplastic syndrome. Second primary malignancies were reported in three patients in the ELd group, and included basal cell carcinoma, myelodysplastic syndrome and squamous cell carcinoma of skin. There were no second primary malignancies in the Ld group. The occurrence of second primary malignancies was generally consistent with previous studies with lenalidomide.^5, 6, 7^ Four patients (13%) in the ELd group reported infusion reactions, which were pyrexia (four patients), asthenia (one patient), chills (one patient) and headache (one patient). All were grade 1 and none led to discontinuation of study medication.

Infections occurred in 81% (25/31) of the ELd group and 79% (23/29) of the Ld group (Table 1). There were no infection-related deaths. Pneumonia was reported in 29% (9/31) of the ELd group compared with 7% (2/29) of the Ld group. Patients with pneumonia in the ELd group were 57–79 years old and experienced grade 2 or 3 pneumonia with onset ranging from study days 10 to 870, 4–27 days from the last dose of elotuzumab, with a duration of 7–31 days. The two patients with pneumonia in the Ld group were 47 and 78 years old, respectively, and experienced grades 2 and 3 pneumonia with onset on study days 155 and 173, with a duration of 22 and 6 days. Although the limited numbers in this analysis make interpretation of this finding difficult, there were no specific factors (for example, patient age, study day or persistence of the infection) associated with pneumonia in patients enrolled from Japan. All cases of pneumonia were reported as serious AEs, but were manageable and resolved by elotuzumab omission or treatment with antibiotics. No patients discontinued elotuzumab due to pneumonia.

In conclusion, Japanese patients treated with ELd experienced prolonged progression-free survival, consistent with the international ELOQUENT-2 population, with a similar overall response rate to the international ELd treatment group.^3^ AEs were manageable and the safety profile was similar to the global study. Although median overall survival has not been reached, preliminary evaluation suggests a benefit with elotuzumab. These results suggest that elotuzumab is a feasible new treatment option in Japanese patients with RRMM, with potential to provide durable responses.

## Figures and Tables

**Figure 1:**
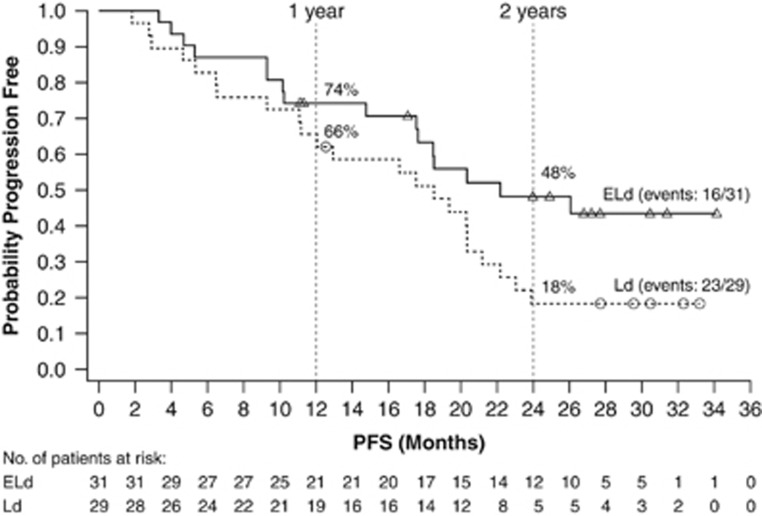
PFS. Abbreviations: ELd, elotuzumab plus lenalidomide and dexamethasone; Ld, lenalidomide and dexamethasone; PFS, progression-free survival.

**Table 1:** Summary of infections

*Parameter*	*ELd (*n=*31)*	*Ld (*n=*29)*
Infection-associated deaths	0	0
Infection (any grade)	25 (81)	23 (79)
Infection (grades 3–4)	12 (39)	5 (17)
Exposure-adjusted infections, per 100 person-years	172.6	183.4
Discontinuation due to infection	2 (6)	0
Serious infections (any grade)	14 (45)	6 (21)
Median time to onset of first infection, months	3.4	3.7
Median duration of first infection, days	15.0	9.0
Pneumonia	9 (29)	2 (7)
Exposure-adjusted pneumonia, per 100 person-years	16.7	4.5

Abbreviations: ELd, elotuzumab plus lenalidomide and dexamethasone; Ld, lenalidomide and dexamethasone.

Data reported as *n* (%) unless indicated otherwise.
