# Supplementary material for: Randomized phase 3 study of elotuzumab for relapsed or refractory multiple myeloma: ELOQUENT-2 Japanese patient subanalysis
Source: Blood Cancer J. 2017 Mar 10;7(3):e540–. doi: 10.1038/bcj.2017.18 (PMC5380903; doi:10.1038/bcj.2017.18)
Supplement: Supplementary Appendix [file bcj201718x1.docx]

**Supplementary Appendix**

This appendix has been provided by the authors to give readers additional information about their work.

Supplement to Suzuki K, et al. Randomized phase 3 study of elotuzumab for relapsed or refractory multiple myeloma: ELOQUENT-2 Japanese patient subanalysis.

**Additional Methods**

**Study design and treatments**

ELOQUENT-2 was conducted in compliance with the Japanese Ministerial Ordinance of Good Clinical Practice based on paragraph 3, Article 14 of the Japanese Pharmaceutical Affairs Law, or International Conference on Harmonisation of Technical Requirements for Registration of Pharmaceuticals for Human Use in Japan, the United States and the European Union. Details of the main study design have been published.^1^

In brief, ELOQUENT-2 was a phase 3, open-label, multicenter trial that enrolled patients between June 2011 and November 2012 (NCT01239797). Patients were randomized 1:1 to elotuzumab plus lenalidomide and dexamethasone (ELd) or lenalidomide and dexamethasone (Ld) and received treatment in 28-day cycles until disease progression, unacceptable toxicity or withdrawal of consent. Patients in the ELd group received elotuzumab (10 mg/kg intravenously) on days 1, 8, 15 and 22 during the first two cycles, and then on days 1 and 15 from the third cycle onwards. Lenalidomide (25 mg/day orally) was given on days 1 through 21, and dexamethasone (40 mg orally) was given on days without elotuzumab or as a split dose (28 mg orally plus 8 mg intravenously) on the day of elotuzumab administration. Patients in the Ld group received lenalidomide (25 mg orally) on days 1 through 21, and dexamethasone (40 mg orally) on days 1, 8, 15 and 22. All patients received premedication before elotuzumab administration to mitigate infusion reactions.

**Study population**

Adult patients with relapsed or refractory multiple myeloma, one to three previous lines of therapy and documented progression after the last line of treatment were enrolled at 18 investigational sites in Japan. All patients had creatinine clearance ≥30 ml/min (as measured by 24-hour urine collection or estimated by the Cockcroft-Gault formula). Prior lenalidomide was permitted, subject to restrictions.

**Assessments**

Efficacy endpoints were centrally assessed by a blinded independent review committee using the criteria of the European Society for Blood and Marrow Transplantation.^2^ The uniform response criteria of the International Myeloma Working Group were used for the evaluation of stringent complete response and very good partial response.^3^ Tumor response was assessed every 4 weeks until progressive disease, death or withdrawal of consent. Overall survival (OS) was a key secondary endpoint. Survival was assessed every 12 weeks after disease progression.

**Statistical analysis**

The type I error rates (two-sided) were 4.5% for progression-free survival (PFS) and 0.5% for overall response rate (ORR).^1^ Interim analysis of PFS was scheduled to be performed when at least 70% of the required events had been observed, and after a minimum follow-up of 2 years. Odds ratio for ORR was stratified by β_2_ microglobulin (<3.5 mg/l vs ≥3.5 mg/l), number of prior lines of therapy (1 vs 2 or 3) and prior immunomodulatory drug (none vs prior thalidomide only vs other) at randomization. Data cut-off, excluding overall survival (OS), was October 2014; interim analysis of OS data was based on data cut-off of October 2015.

**References**

1. Lonial S, Dimopoulos M, Palumbo A, White D, Grosicki S, Spicka I *et a*l. Elotuzumab therapy for relapsed or refractory multiple myeloma. *N Engl J Med* 2015; **373**: 621–631.
2. Bladé J, Samson D, Reece D, Apperley J, Bjorkstrand B, Gahrton G *et al*. Criteria for evaluating disease response and progression in patients with multiple myeloma treated by high-dose therapy and haemopoietic stem cell transplantation. Myeloma Subcommittee of the EBMT. European Group for Blood and Marrow Transplant. *Br J Haematol* 1998; **102**: 1115–1123.
3. Durie BG, Harousseau JL, Miguel JS, Blade J, Barlogie B, Anderson K *et al.* International uniform response criteria for multiple myeloma. *Leukemia* 2006; **20**: 1467–1473.

**Supplementary Table S1.** Baseline demographics, disease characteristics and treatment summary (*N*=60)

| Characteristic | ELd  (*n*=31) | Ld  (*n*=29) |
| --- | --- | --- |
| Age (years) |  |  |
| Median (range) | 69 (45–80) | 66 (47–84) |
| ≥65 years | 21 (68) | 16 (55) |
| ISS disease stage |  |  |
| I | 23 (74) | 17 (59) |
| II | 7 (23) | 12 (41) |
| III | 1 (3) | 0 |
| Cytogenetics (FISH) |  |  |
| del(17p) |  |  |
| Yes | 6 (19) | 8 (28) |
| No | 24 (77) | 21 (72) |
| Not reported | 1 (3) | 0 |
| t(4;14) |  |  |
| Yes | 4 (13) | 7 (24) |
| No | 26 (84) | 22 (76) |
| Not reported | 1 (3) | 0 |
| 1q21 |  |  |
| Yes | 12 (39) | 11 (38) |
| No | 18 (58) | 18 (62) |
| Not reported | 1 (3) | 0 |
| Prior regimens |  |  |
| Median (range) | 1 (1–3) | 2 (1–3) |
| Prior therapies^a^ |  |  |
| Bortezomib | 21 (68) | 16 (55) |
| Melphalan | 28 (90) | 26 (90) |
| Thalidomide | 6 (19) | 6 (21) |
| Response to the most recent line of therapy |  |  |
| Refractory | 11 (35) | 13 (45) |
| Relapsed | 20 (65) | 16 (55) |
| Prior SCT | 11 (35) | 13 (45) |
| Number of treatment cycles |  |  |
| Median (range) | 22 (2–38) | 16 (3–36) |
| Patients remaining on treatment | 12 (39) | 5 (17) |
| Relative dose intensity (≥90%) |  |  |
| Elotuzumab | 26 (84) | – |
| Lenalidomide | 10 (32) | 13 (45) |
| Dexamethasone | 10 (32) | 11 (38) |
| Data reported as n (%) unless indicated otherwise. Abbreviations: ELd, elotuzumab plus lenalidomide and dexamethasone; FISH, fluorescence in situ hybridization; ISS, International Staging System; Ld, lenalidomide and dexamethasone; SCT, stem cell transplantation. ^a^Prior lenalidomide was permitted if the best response was a partial response or better and patients were not refractory to prior lenalidomide treatment; patients could not receive more than nine cycles of lenalidomide and had to have at least 9 months between the last dose of lenalidomide and progression. | | |

**Supplementary Table S2.** Best responses

| Response | ELd  (*n*=31) | Ld  (*n*=29) |
| --- | --- | --- |
| Overall response rate^a^ | 26 (84) | 25 (86) |
| Odds ratio (95% CI) | 0.68 (0.16–2.90) | |
| Best response |  |  |
| Stringent complete response | 0 | 1 (3) |
| Complete response | 0 | 3 (10) |
| Very good partial response | 8 (26) | 6 (21) |
| Partial response | 18 (58) | 15 (52) |
| Minimal response | 2 (6) | 0 |
| Stable disease | 3 (10) | 2 (7) |
| Progressive disease | 0 | 2 (7) |
| Data reported as n (%) unless indicated otherwise. Abbreviations: CI, confidence interval; ELd, elotuzumab plus lenalidomide and dexamethasone; Ld, lenalidomide and dexamethasone. ^a^Partial response or better. | | |

**Supplementary Table S3.** Adverse events

| **Event, *n* (%)** | **ELd**  **(*n*=31)** | | **Ld**  **(*n*=29)** | |
| --- | --- | --- | --- | --- |
|  | **Any grade** | **Grade 3 or 4** | **Any grade** | **Grade 3 or 4** |
| Any AEs | 31 (100) | 29 (94) | 29 (100) | 22 (76) |
| **Common hematologic toxic effect^a^** | | | | |
| Lymphopenia | 31 (100) | 26 (84) | 29 (100) | 17 (59) |
| Leukopenia | 31 (100) | 7 (23) | 22 (76) | 3 (10) |
| Anemia | 29 (94) | 6 (19) | 28 (97) | 4 (14) |
| Neutropenia | 29 (94) | 11 (35) | 26 (90) | 10 (34) |
| Thrombocytopenia | 27 (87) | 1 (3) | 25 (86) | 4 (14) |
| **Common non-hematologic AEs^b^** | | | | |
| Nasopharyngitis | 15 (48) | 0 | 15 (52) | 0 |
| Constipation | 14 (45) | 0 | 12 (41) | 0 |
| Pyrexia | 13 (42) | 0 | 8 (28) | 1 (3) |
| Rash | 11 (35) | 0 | 7 (24) | 0 |
| Diarrhea | 9 (29) | 0 | 7 (24) | 0 |
| Pneumonia | 9 (29) | 6 (19) | 2 (7) | 1 (3) |
| Peripheral edema | 8 (26) | 0 | 7 (24) | 1 (3) |
| Dysgeusia | 7 (23) | 0 | 5 (17) | 0 |
| Muscle spasms | 7 (23) | 0 | 3 (10) | 0 |
| Contusion | 7 (23) | 0 | 3 (10) | 0 |
| Decreased appetite | 6 (19) | 2 (6) | 7 (24) | 4 (14) |
| Cataract | 6 (19) | 6 (19) | 6 (21) | 4 (14) |
| Malaise | 6 (19) | 0 | 5 (17) | 0 |
| Bronchitis | 5 (16) | 0 | 8 (28) | 1 (3) |
| Upper respiratory tract inflammation | 5 (16) | 0 | 5 (17) | 0 |
| Back pain | 5 (16) | 1 (3) | 4 (14) | 0 |
| Stomatitis | 5 (16) | 0 | 3 (10) | 0 |
| Nausea | 5 (16) | 1 (3) | 2 (7) | 0 |
| Weight decreased | 5 (16) | 1 (3) | 2 (7) | 0 |
| Insomnia | 4 (13) | 0 | 11 (38) | 0 |
| Peripheral neuropathy | 4 (13) | 0 | 6 (21) | 1 (3) |
| Hiccups | 4 (13) | 0 | 5 (17) | 0 |
| Somnolence | 4 (13) | 1 (3) | 2 (7) | 0 |
| Cough | 4 (13) | 0 | 2 (7) | 0 |
| Hepatic function abnormal | 4 (13) | 0 | 2 (7) | 0 |
| Urticaria | 4 (13) | 0 | 2 (7) | 0 |
| Hypoesthesia | 4 (13) | 0 | 1 (3) | 0 |
| Dehydration | 4 (13) | 0 | 0 | 0 |
| Fatigue | 3 (10) | 0 | 5 (17) | 0 |
| Peripheral sensory neuropathy | 3 (10) | 1 (3) | 4 (14) | 0 |
| Hyperglycemia | 3 (10) | 2 (6) | 3 (10) | 3 (10) |
| Hypokalemia | 3 (10) | 2 (6) | 3 (10) | 1 (3) |
| Dysphonia | 3 (10) | 0 | 3 (10) | 0 |
| Pruritus | 2 (6) | 0 | 3 (10) | 0 |
| Increased alanine aminotransferase | 1 (3) | 0 | 5 (17) | 2 (7) |
| Gastroenteritis | 1 (3) | 0 | 3 (10) | 0 |
| Upper respiratory tract infection | 1 (3) | 0 | 3 (10) | 0 |
| Hypogeusia | 0 | 0 | 3 (10) | 0 |

Abbreviations: AE, adverse event; ELd, elotuzumab plus lenalidomide and dexamethasone; Ld, lenalidomide and dexamethasone. ^a^Data are based on worst toxicity grade recorded in laboratory testing. ^b^Reported in ≥10% of patients**.**

**Supplementary Table S4.** Serious adverse events in ≥5% of patients

| Adverse event,^a^ *n* (%) | ELd  (*n*=31) | | Ld  (*n*=29) | |
| --- | --- | --- | --- | --- |
|  | Any grade | Grade 3^b^ | Any Grade | Grade 3^b^ |
| Pneumonia | 9 (29) | 6 (19) | 2 (7) | 1 (3) |
| Cataract | 4 (13) | 4 (13) | 3 (10) | 3 (10) |
| Decreased appetite | 2 (6) | 2 (6) | 1 (3) | 1 (3) |
| Plasma cell myeloma | 2 (6) | 2 (6) | 0 | 0 |
| Deep vein thrombosis | 0 | 0 | 2 (7) | 2 (7) |

Abbreviations: ELd, elotuzumab plus lenalidomide and dexamethasone; Ld, lenalidomide and dexamethasone. ^a^Medical Dictionary for Regulatory Activities preferred term. ^b^No grade 4 or 5 events among the AEs that occurred in ≥5% of patients.
